# Culturing Articular Cartilage Explants in the Presence of Autologous Adipose Tissue Modifies Their Inflammatory Response to Lipopolysaccharide

**DOI:** 10.1155/2020/8811001

**Published:** 2020-11-12

**Authors:** Wendy Pearson, Anna E. N. Garland, Ashley Nixon, John P. Cant, Mark B. Hurtig

**Affiliations:** ^1^Department of Animal Biosciences, University of Guelph, Guelph, ON, Canada N1G 2W1; ^2^Department of Clinical Studies, University of Guelph, Guelph, ON, Canada N1G 2W1

## Abstract

The purpose of the current study was to explore the effect of autologous adipose tissue on cartilage responses to lipopolysaccharide (LPS). We hypothesized that LPS elicits an inflammatory response in cartilage, and that response is augmented in the presence of adipose tissue. Furthermore, we hypothesized that this augmented inflammatory response is due, at least in part, to increased exposure of cartilage to adipose tissue-derived c3a. Porcine cartilage explants from market-weight pigs were cultured in the presence or absence of autologous adipose tissue for 96 hours, the final 48 hours of which they were stimulated with LPS (0 or 10 *μ*g/mL). Adipose tissue explants were also cultured alone, in the presence or absence of LPS. Media from all cartilage treatments was assayed for c3a/c3a des Arg, PGE_2_, GAG, and NO, and the viability of cartilage tissue was determined by differential fluorescent staining. Media from adipose tissue explants was assayed for c3a/c3a des Arg and PGE_2_. LPS produced a significant increase in PGE_2_, GAG, and NO production when cartilage was cultured in the absence of adipose tissue. Coculture of adipose tissue prevented a significant increase in PGE_2_ in cartilage explants. There was no effect of adipose tissue on LPS-induced GAG or NO, but the presence of adipose tissue significantly reduced cell viability in LPS-stimulated cartilage explants. Adipose tissue explants from lean animals reduced inflammatory responses of cartilage to LPS via a c3a/c3a des Arg-independent mechanism and were associated with a significant decline in cell viability. Thus, contrary to our hypothesis, adipose tissue from lean animals does not augment the inflammatory response of cartilage to stimulation by LPS. The mechanism of modulatory effects of adipose tissue on LPS-induced increase in PGE_2_ and decline in chondrocyte viability requires further research but appears to have occurred via a mechanism that is independent of adipocentric c3a/c3a des Arg.

## 1. Introduction

Adipose tissue, once regarded as an indolent vehicle for fuel storage, is now known to play vital roles in integrating whole-body fuel metabolism, including mobilization and combustion of fuels and energy homeostasis. Crucial missing links in the involvement of adipose tissue in the integration of organism energy metabolism were the discoveries of its multifaceted endocrine and paracrine functions [1]. Awareness of the complex array of signalling molecules (including leptin, cytokines, and components of the alternative complement pathway) produced by adipose tissue has broadened our understanding of its highly complex and diverse roles [2, 3]. The characteristic of adipose tissue in overweight and obese individuals is a maladaptive profile of secreted adipokines and cytokines emulating that of nonobese individuals suffering from a systemic inflammatory condition [4]. This proinflammatory endocrinological state can be implicated, at least in part, in the abundance of chronic inflammatory diseases frequently comorbid in obese and overweight individuals [5]. Osteoarthritis (OA) is one such condition that is highly prevalent in obese individuals, and obesity-associated OA is considered the fifth component of metabolic syndrome in humans [6, 7]. Obesity is estimated to affect as much as half the world's adult population by 2030, if prevalence continues in the current trend [8]; OA of weight-bearing and non-weight-bearing joints makes a major contribution to the total economic cost of overweight and obesity in humans and is the 11^th^ highest contributor to global disability [9].

While the phenotypic interactions between OA and obesity are evident, the underlying pathology that drives these interactions is less so. However, it is known that adipose tissue is an important source of complement proteins such as anaphylatoxin “c3a” [10], which contribute to adipocentric tissue inflammation [10, 11], development of insulin resistance [11, 12], and immune system activation [13]. The anaphylatoxin c3a is a potent inducer of arthritic changes in cartilage [14] and provokes overproduction of proinflammatory compounds including eicosanoids (especially PGE_2_) [15]. It has been proposed that c3a derived from adipose tissue may contribute to obesity-associated inflammatory disorders [10].

Thus, the purpose of the current study was to determine responses of cartilage to the proinflammatory stimulus lipopolysaccharide (LPS) in the presence or absence of autologous adipose tissue. We hypothesize that LPS elicits an inflammatory response in cartilage, and that response is augmented in the presence of adipose tissue. Furthermore, we hypothesize that this augmented inflammatory response is due, at least in part, to increased exposure of cartilage to adipose tissue-derived c3a.

## 2. Materials and Methods

### 2.1. Tissue Collection

Front limbs inclusive of the carpal joint from market-weight pigs (*n* = 16) slaughtered for human consumption were obtained from a federally inspected abattoir and transported for approximately 1 h on ice to the laboratory. The intercarpal joint was opened, and cartilage (C) explants (4 mm; 15.9 ± 0.26 mg/explant) were obtained. Adipose tissue (A) was excised from the upper leg, and A explants (27.5 ± 0.94 mg/explant) were prepared using surgical scissors and scalpel. C and A explants were washed three times in Dulbecco's Modified Eagle's Medium (DMEM) with sodium bicarbonate.

### 2.2. Experimental Design

**Figure 1:**
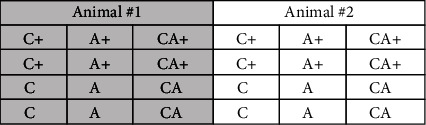
Layout of a tissue culture plate. A total of 8 plates were used, 2 animals per plate, for an “*n*” of 16 animals. C = cartilage explant; A = adipose tissue explant; + = inclusion of LPS (10 *μ*g/mL).

For each animal, cartilage explants were arranged into 24-well tissue culture plates (2 per well per animal) in the presence or absence of a single A explant. Single A explants were also cultured in the absence of C. Plates were incubated at 37°C, 7% CO_2_ in DMEM supplemented with amino acids and antibiotics for a total of 120 h, with media changes occurring every 24 h [16]. *Escherichia coli* serotype O128:B12 LPS (0 or 10 *μ*g/mL; “+” indicates inclusion of LPS) was added to wells for the final 48 h of culture, such that each treatment was maintained either in the presence or in the absence of LPS. Media from the final 48 h of culture was collected each day into microcentrifuge tubes containing 10 *μ*g indomethacin and frozen at -20°C until analysis (approximately one month).  Figure 1 illustrates the experimental design.

### 2.3. Sample Analysis

Tissue culture media for explant wells A, A+, C, C+, CA, and CA+ was analyzed from 8 animals for PGE_2_ and c3a/c3a des Arg. Tissue culture media from C, C+, CA, and CA+ wells from all 16 animals was also analyzed for glycosaminoglycan (GAG) and nitric oxide (NO). Cartilage explants were stained with calcein-AM and ethidium homodimer-1 for an estimate of cell viability from all 16 animals.

All spectrophotometry and fluorescence readings were obtained from a microplate reader (1420 Victor 2, PerkinElmer; Fusion *α*, PerkinElmer). All chemical reagents were purchased from Sigma-Aldrich (Mississauga, ON, Canada) unless otherwise stated.

#### 2.3.1. Cell Viability

The viability of cells within cartilage explants was determined using a calcein-AM (C-AM)/ethidium homodimer-1 (EthD-1) cytotoxicity assay kit (ThermoFisher; Catalog #L3224) modified for use in cartilage explants [16]. The C-AM and EthD-1 were mixed in sterile distilled water at concentrations of 4 and 8 *μ*M, respectively. Explants were arranged into a 96-well microtitre plate (one explant per well) and incubated in 200 *μ*L of C-AM/EthD-1 solution for 40 min at room temperature. The microplate reader was set to scan each well, beginning at the bottom, using 10 horizontal steps at each of 3 vertical displacements set to 0.1 mm apart. The C-AM and EthD-1 fluorescence in explants were obtained using excitation/emission filters of 485/530 and 530/685, respectively.

#### 2.3.2. PGE_2_

PGE_2_ concentration of tissue culture media samples was determined using a commercially available ELISA kit (Enzo Life Sciences; Catalog #ADI-900-001). Samples were thawed to room temperature and randomly loaded onto antibody-coated 96-well microtitre plates according to kit instructions. A best-fit 4^th^ order polynomial standard curve was developed for each plate (*R*^2^ > 0.99), and these equations were used to calculate PGE_2_ concentrations for samples from each plate.

#### 2.3.3. c3a/c3a des Arg

c3a/c3a des Arg concentration (a stable metabolite of c3a) of tissue culture media samples was determined using a commercially available ELISA kit (Enzo Life Sciences; Catalog # ADI-900-058). Samples were thawed to room temperature and randomly loaded onto antibody-coated 96-well microtitre plates according to kit instructions. A best-fit 4^th^ order polynomial standard curve was developed for each plate (*R*^2^ > 0.99), and these equations were used to calculate c3a/c3a des Arg concentrations for samples from each plate.

#### 2.3.4. NO

Nitrite (NO_2_^−^), a stable oxidation product of NO, was analyzed by the Griess Reaction. Undiluted media samples were added to 96-well microtitre plates. Sulfanilamide (0.01 g/mL) and N-(1)-napthylethylene diamine hydrochloride (1 mg/mL) dissolved in phosphoric acid (0.85 g/L) were added to all wells, and absorbance was read within 5 min at 530 nm. Sample absorbance was compared to a sodium nitrite standard curve. A best-fit linear regression equation was developed from standard curves from each plate (*R*^2^ ≥ 0.99), and these equations were used to calculate nitrite concentrations for samples from each plate.

#### 2.3.5. GAG

GAG concentration of media samples was determined using a 1,9-dimethyl methylene blue spectrophotometric assay. Samples were thawed to room temperature and randomly loaded onto a 96-well microtitre plate with a dilution factor of 2 : 13 using dilution buffer (410 mg sodium acetate and 50 *μ*L Tween 20 in 100 mL double-distilled water). Guanidine hydrochloride (275 mg/mL) and DMB reagent (200 *μ*L) were added to each well. Plates were read at absorbance 530 nm and compared to a bovine chondroitin sulfate standard. A best-fit linear standard curve was developed for each plate (*R*^2^ > 0.98), and these equations were used to calculate GAG concentration for samples on each plate.

### 2.4. Data Analysis

The experimental unit is “pig.” Pigs and treatments were run in duplicate, which were analyzed individually, and the mean of duplicates was used in the statistical analysis. Data from the final 48 h of culture are presented as mean ± SD per mg of cartilage tissue for C, C+, CA, and CA+ treatments. Data from the final 48 h of culture of A and A+ explants are presented as mean ± SD per mg of adipose tissue. Time “0” is the baseline sample after the first 48 h of culture prior to the addition of LPS. Each animal represents a single observation (i.e., experimental unit). To compare effects of treatments over time, PGE_2_, GAG, and NO were analyzed using a 2-way RM ANOVA (SigmaPlot; version 12) with respect to time and treatment. Cell viability data were analyzed using a 1-way ANOVA with respect to treatment. When a significant *F*-ratio was obtained, the Holm-Sidak post hoc test was used to detect significantly different means. Significance was accepted with *p* < 0.05.

## 3. Results and Discussion

### 3.1. Cell Viability

**Figure 2:**
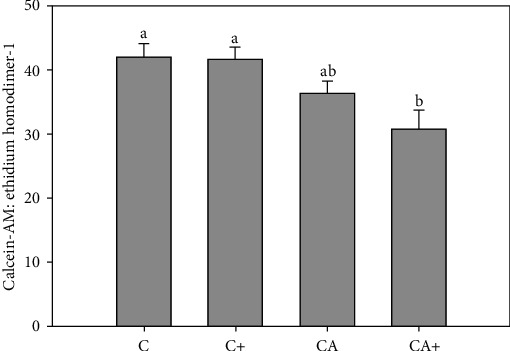
Ratio of calcein-AM to ethidium homodimer-1 in cartilage explants (C) in the presence or absence of adipose tissue explants (A) and/or LPS (+). Data represent duplicate samples from 16 market-weight pigs. Different letters denote significantly different means (*p* < 0.05).

CA+ explants (30.8 ± 13.9) had significantly lower viability compared with C (42.0 ± 10.1) and CA (36.4 ± 8.3) explants (Figure 2). The viability of CA explants was nonsignificantly reduced compared with C and C+ (41.7 ± 9.3) explants and was not significantly different from CA+ explants.

### 3.2. c3a/c3a des Arg

**Figure 3:**
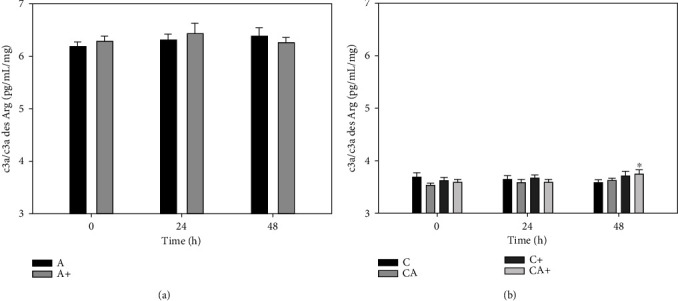
c3a/c3a des Arg production by adipose tissue explants (a) and cartilage explants (C) cultured in the presence or absence of adipose tissue explants (A) (b) with or without LPS (+; 10 *μ*g/mL). Data represent duplicate samples from 8 market-weight pigs. ^∗^Denotes significant change from 0 h within a single treatment group (*p* < 0.05).

There was no change in c3a/c3a des Arg in A explants at either 24 h (6.31 ± 0.36 pg/mL/mg) or 48 h (6.38 ± 0.48 pg/mL/mg) compared with 0 h (6.19 ± 0.24 pg/mL/mg) (Figure 3(a)). LPS stimulation of A+ explants did not produce any change in c3a/c3a des Arg at either 24 h (6.42 ± 0.60 pg/mL/mg) or 48 h (6.27 ± 0.29 pg/mL/mg) compared with 0 h (6.28 ± 0.31 pg/mL/mg). The c3a/c3a des Arg concentration was not significantly different between A+ and A explants at any time point; however, c3a/c3a des Arg was significantly higher in A and A+ explants than in C, C+, CA, and CA+ explants at all timepoints.

There was no significant change in c3a/c3a des Arg production in C or CA explants over the final 48 h of culture (Figure 3(b)). The c3a/c3a des Arg production in CA+ explants was significantly increased at 48 h (3.74 ± 0.27 pg/mL/mg) compared with 0 h (3.59 ± 0.14 pg/mL/mg). There was no significant change in c3a/c3a des Arg production in C+ explants, and there were no significant differences in c3a/c3a des Arg production between C, C+, CA, or CA+ at any time point.

### 3.3. PGE_2_

**Figure 4:**
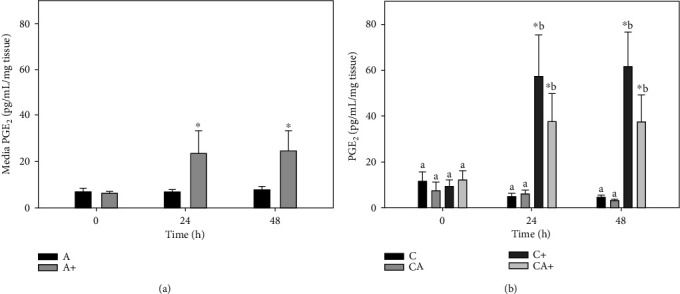
Prostaglandin E_2_ (PGE_2_) production by adipose tissue explants (a) and cartilage explants (C) cultured in the presence or absence of adipose tissue explants (A) (b) with or without LPS (+; 10 *μ*g/mL). Data represent duplicate samples from 8 market-weight pigs. ^∗^Denotes significant change from 0 h within a single treatment group. Lowercase letters denote significantly different means at a single time point (*p* < 0.05).

There was no change in PGE_2_ in A explants at either 24 h (6.8 ± 2.9 pg/mL/mg) or 48 h (7.7 ± 3.8 pg/mL/mg) compared with 0 h (6.8 ± 4.6 pg/mL/mg) (Figure 4(a)). LPS stimulation of A+ explants produced a significant increase in PGE_2_ at 24 h (23.4 ± 29.7 pg/mL/mg) and 48 h (24.6 ± 25.4 pg/mL/mg) compared with 0 h (6.0 ± 2.7 pg/mL/mg). PGE_2_ concentration was significantly higher in A+ explants than A explants at 24 and 48 h.

There was no significant change in PGE_2_ production in C or CA explants over the final 48 h of culture (Figure 4(b)). PGE_2_ production in C+ explants was significantly increased at 24 h (57.5 ± 52.8 pg/mL/mg) and 48 h (61.6 ± 44.8 pg/mL/mg) compared with 0 h (9.3 ± 9.1 pg/mL/mg). The increase in PGE_2_ in CA+ explants at 24 h (37.6 ± 36.4 pg/mL/mg) and 48 h (37.5 ± 35.4 pg/mL/mg) relative to 0 h (12.0 ± 12.9 pg/mL/mg) was not significant. At 24 and 48 h, C+ explants had significantly higher PGE_2_ than C and CA explants but were not higher than CA+ explants.

### 3.4. NO

**Figure 5:**
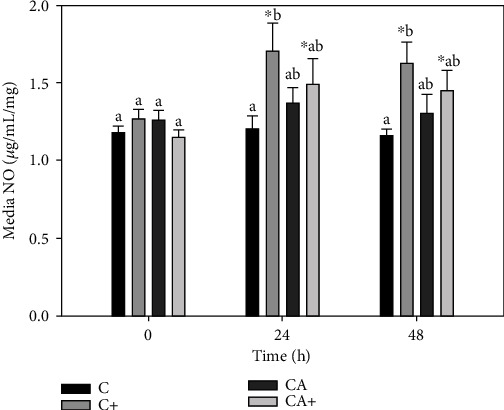
Nitric oxide (NO) production by cartilage explants (C) cultured in the presence or absence of adipose tissue explants (A) and/or LPS (+; 10 *μ*g/mL). Data represent duplicate samples from 16 market-weight pigs. ^∗^Denotes significant change from 0 h within a single treatment group. Lowercase letters denote significantly different means at a single time point (*p* < 0.05).

There was no significant change in NO production in C or CA explants over the final 48 h of culture (Figure 5). NO production in C+ explants was significantly increased at 24 h (1.7 ± 0.5 *μ*g/mL/mg) and 48 h (1.6 ± 0.4 *μ*g/mL/mg) compared with 0 h (1.3 ± 0.2 *μ*g/mL/mg). There was also a significant increase in NO in CA+ explants at 24 (1.5 ± 0.5 *μ*g/mL/mg) and 48 h (1.4 ± 0.38 *μ*g/mL/mg) relative to 0 h (1.2 ± 0.1 *μ*g/mL/mg). At 24 and 48 h, C+ explants had significantly higher NO than C explants but were not higher than CA or CA+ explants.

### 3.5. GAG

**Figure 6:**
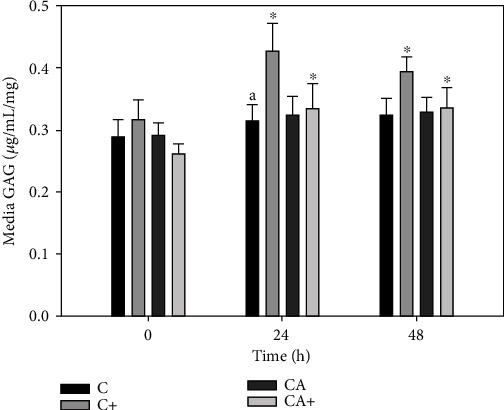
Glycosaminoglycan (GAG) release from cartilage explants (C) cultured in the presence or absence of adipose tissue explants (A) and/or LPS (+; 10 *μ*g/mL). Data represent duplicate samples from 16 market-weight pigs. ^∗^Denotes significant change from 0 h within a single treatment group (*p* < 0.05).

There was no significant change in GAG release from C or CA explants over the final 48 h of culture (Figure 6). GAG release in C+ explants was significantly increased at 24 h (0.42 ± 0.33 *μ*g/mL/mg) and 48 h (0.40 ± 0.07 *μ*g/mL/mg) compared with 0 h (0.32 ± 0.09 *μ*g/mL/mg). There was also a significant increase in GAG release from CA+ explants at 24 h (0.34 ± 0.11 *μ*g/mL/mg) and 48 h (0.34 ± 0.09 *μ*g/mL/mg) relative to 0 h (0.26 ± 0.05 *μ*g/mL/mg). There were no significant differences in GAG release among groups at any individual time point.

## 4. Discussion

The current study explored the effect of autologous adipose tissue on cartilage explant responses to inflammatory stimulation with LPS. The main findings are that, when stimulated with LPS *in vitro*, adipose tissue explants significantly increase PGE_2_ but not c3a/c3a des Arg production. LPS stimulation of cartilage explants increased PGE_2_ without reducing cell viability, but this increase was not significant when LPS-stimulated cartilage explants were cocultured with adipose tissue, and cell viability was significantly reduced. Contrary to our hypothesis, coculturing of cartilage explants with adipose tissue explants appeared to blunt the inflammatory response of cartilage to LPS rather than augment it. Similar findings have been reported by others who showed that coculturing of articular cartilage and meniscus with infrapatellar fat pad afforded measurable protection against structural breakdown of the tissue [17].

The lack of c3a response of A+ explants to LPS was unexpected, as LPS activates the lectin complement pathway resulting in enhanced production of c3 by adipocytes [10]. Our results may have occurred due to further processing of c3 to c5a and c5b, the latter of which comprises the core of the c5b-9 terminal complement complex (membrane attack complex) [18]. This complex plays a crucial role in innate immunity, primarily by activating transcription factors and signal transduction pathways which lead to cell lysis [18]. Further processing of c3a to c5b may explain the lack of increase in LPS-induced c3a in the current study, in addition to the significant decline in the viability of chondrocytes we observed within cartilage explants stimulated with LPS in the presence of adipose tissue. To characterize the effect of exogenous LPS on adipose tissue production and activation of complement proteins *in vitro*, future studies should focus on isolation and quantification of c5b-9. Indeed, c5b-9 may be critically important to characterize in an in vitro system as utilized in the current study, as part of its role in adipocentric inflammation is the homing of immune cells into adipose tissue [19], a role it is incapable of fulfilling in an isolated explant system.

While our study does not provide evidence for a significant role of adipocentric c3a in response of cartilage to LPS stimulation, the adipose tissue explants were metabolically active as evidenced by their significant PGE_2_ production in response to LPS. Unlike cartilage, which possesses a decidedly unicellular profile of chondrocytes, adipose tissue contains a variety of cell types including adipocytes, neutrophils, T-lymphocytes, mast cells, and macrophages [20]. It is likely that LPS-induced PGE_2_ from A+ explants in the current study likely arose, at least in part, from macrophages [21]. There are 2 distinct phenotypes of macrophages in adipose tissue, termed “M1” and “M2” [22]. Both phenotypes are present in adipose tissue; the former predominates in obese adipose tissue and produces proinflammatory compounds such as IL-6 and TNF-*α* [23, 24] and participates in the development of insulin resistance and inflammation in obese individuals [24]. The latter “M2” phenotype predominates in adipose tissue from lean individuals and functions to promote tissue repair and inhibit M1 macrophages by producing anti-inflammatory compounds such as IL-10 [22, 23]. Reversible polarization of M2 macrophages to the M1 phenotype can occur in response to elevated LPS in the local microenvironment [25] and may have contributed to the production of LPS-induced PGE_2_ in A+ explants in the current study. The local increase in PGE_2_ could then, in turn, facilitate return to the M2 phenotype [26], implicating M2 macrophages in tissue repair [27] and inflammation resolution [28]. To our knowledge, the literature does not yet provide a clear indication of how much time is required for macrophage polarization (in either direction) *in vitro*, and further research is required to understand the role that macrophage polarization may have played in the current study. Nonetheless, since the adipose tissue used in the current study was obtained from market-weight pigs (i.e., not obese), it is likely that our explants contained macrophages of the predominately M2 phenotype which probably directed, to some extent, the response of cartilage explant to LPS. Future studies should attempt to characterize the relative predominance of M1 and M2 phenotypes, based on their production of pro- and anti-inflammatory products, respectively.

A high M2:M1 macrophage phenotype within our adipose tissue explants may have played a role in the lack of significant adipocentric provocation of LPS-induced PGE_2_ production in CA+ explants. LPS predictably induces PGE_2_ from cartilage explants [16, 29], and this was also observed in the current study. And while PGE_2_ concentrations between C+ and CA+ were not different at any time point, coculture of cartilage with adipose tissue prevented a significant effect of LPS on PGE_2_ production in CA+ explants. M2 macrophages produce the anti-inflammatory cytokine IL-4 [30], which has an inhibitory effect on LPS-induced PGE_2_ in uterine cells [31] and rheumatoid synovial cells [32], and may have contributed to the blunted PGE_2_ response of CA+ explants in the current study.

The lack of significant effect of adipose explants on LPS-induced GAG loss and NO production in the current study is also consistent with M2 macrophages predominating as the major macrophage phenotype. Others report no direct inhibitory effect of M2 macrophages on IL-1- or TNF-*α*-induced GAG loss or NO production in human cartilage explants [33], consistent with our findings.

We observed a significant decline in chondrocyte viability within explants in the presence of adipose tissue and LPS. The reason for this decline is not known but may involve the profusion of adipokines produced by adipocytes, which includes leptin. Leptin is produced in abundance from adipose tissue [34] and induces apoptosis in cultured chondrocytes [35, 36], which may have contributed to the reduced viability observed in CA+ explants. Adipocytes also produce at least 29 proteins that are involved in oxidative stress [37], a metabolic process that can often lead to increased cell death [38]. The molecular weight of some of these compounds exceeds that which can be expected to infiltrate the joint capsule during an inflammatory event [38], so may not produce a similar decline in cell viability in the *in vivo* condition. Future coculture studies should employ a transwell tissue culture system with a selective membrane cut-off of 100 kDa [38] or use tissue culture media previously conditioned with adipose tissue and filtered to <100 kDa.

## 5. Conclusions

In conclusion, contrary to our hypothesis, autologous adipose tissue explants from market-weight animals does not increase the inflammatory response of cartilage explants to LPS. This may indicate a protective role of adipose tissue against inflammation in nonobese individuals. Future studies into physiological interactions between adipose tissue and cartilage as they relate to cartilage inflammation should compare tissue of lean and obese individuals to better understand how the anti-inflammatory characteristics of lean adipose tissue might be translated to that of obese individuals.

## Data Availability

Data is available by request from the corresponding author.
